# Gut microbiota dysbiosis and the gut–lung axis: links to asthma and its common comorbidities

**DOI:** 10.7717/peerj.20960

**Published:** 2026-03-13

**Authors:** Jianyu Xie, Jianwei Yu

**Affiliations:** Jiangxi University of Traditional Chinese Medicine, Nanchang, China

**Keywords:** Asthma, Gut microbiota, Obesity, Allergic rhinitis, Chronic rhinosinusitis, Gastroesophageal reflux

## Abstract

Asthma is a heterogeneous chronic inflammatory airway disease that frequently coexists with multiple comorbidities, including obesity, allergic rhinitis, chronic rhinosinusitis, and gastroesophageal reflux disease (GERD). These conditions substantially increase disease burden and complicate asthma management. Increasing evidence suggests that gut microbiota dysbiosis represents a shared biological factor linking asthma and its comorbidities through immune and metabolic pathways operating along the gut–lung axis. This narrative review summarizes current knowledge on how alterations in gut microbial composition and function influence immune homeostasis, chronic inflammation, and disease susceptibility in asthma and its common comorbid conditions. Particular emphasis is placed on microbial metabolites such as short-chain fatty acids, early-life immune programming, bidirectional gut–lung immune communication, and emerging microbiota-targeted interventions. Understanding the systemic role of the gut microbiota may provide novel insights into integrated prevention and management strategies for asthma and its comorbidities.

## Introduction

Asthma is a common chronic inflammatory airway disease characterized by variable airflow obstruction and airway hyperresponsiveness, affecting more than 300 million individuals worldwide (Zhang et al., 2025). Despite advances in pharmacological therapy, asthma remains highly heterogeneous with respect to clinical presentation, inflammatory endotypes, and treatment responsiveness (Kaur & Chupp, 2019).

Asthma rarely occurs in isolation and is frequently accompanied by comorbidities such as obesity, allergic rhinitis, chronic rhinosinusitis, and gastroesophageal reflux disease (Althoff et al., 2021). While allergic rhinitis and chronic rhinosinusitis share classical Type 2 immune mechanisms with asthma, obesity and GERD are non-allergic but highly prevalent comorbidities that influence asthma through immunometabolic, mechanical, and inflammatory pathways (Althoff et al., 2021; Huang et al., 2023).

In recent years, environmental and lifestyle factors have gained increasing attention in asthma research. The gut microbiota, a complex microbial ecosystem essential for immune maturation and metabolic homeostasis, has emerged as a critical mediator linking environmental exposures to asthma susceptibility and disease progression (Wang et al., 2021; Belkaid & Hand, 2014). Through the gut–lung axis, intestinal microbes and their metabolites can influence pulmonary immunity, while lung inflammation may reciprocally affect gut microbial composition (Zhang et al., 2025).

Despite rapid growth in this field, an integrated synthesis connecting gut microbiota dysbiosis to asthma and its major comorbidities remains limited (O’Dwyer, Dickson & Moore, 2016). This narrative review aims to summarize current evidence on (i) the role of the gut microbiota in immune homeostasis, (ii) mechanisms underlying gut–lung and lung–gut immune crosstalk, (iii) microbiota-related pathways contributing to asthma comorbidities, and (iv) emerging microbiota-based therapeutic strategies. By adopting a systemic perspective, this review seeks to provide clinicians and researchers with a conceptual framework for understanding asthma as a multi-organ disease influenced by microbial ecology.

## Survey Methods

This study is a narrative (non-systematic) literature review. PubMed (MEDLINE), Web of Science, and Embase were searched for English-language articles published up to August 2025. Search terms included combinations of “asthma,” “gut microbiota” or “microbiome,” “gut–lung axis,” “obesity,” “allergic rhinitis,” “chronic rhinosinusitis,” and “gastroesophageal reflux disease.”

Peer-reviewed human studies (epidemiological investigations, clinical trials, cohort and case–control studies) were included to establish associations and clinical relevance. Relevant animal and *in vitro* studies were incorporated to provide mechanistic insights. Review articles and meta-analyses were also considered. Studies not addressing the gut microbiota in the context of asthma or the specified comorbidities were excluded.

Given the narrative scope, no formal meta-analysis, study flow diagram, or quantitative risk-of-bias assessment was performed. This narrative approach was chosen to allow conceptual integration of mechanistic and clinical evidence rather than quantitative effect-size estimation.

## Asthma and Its Common Comorbidities

### Obesity

Obesity is an independent risk factor for asthma and is associated with a distinct obesity-related asthma phenotype, which is characterized by non–Type 2 inflammation, particularly neutrophilic airway inflammation, with reduced corticosteroid responsiveness (Huang et al., 2023; Miethe et al., 2018; Turnbaugh et al., 2009; Cani et al., 2008; Cani, 2013). Importantly, obesity-related asthma remains heterogeneous, and additional sub-phenotypes likely exist but are less well characterized (Miethe et al., 2018).

Beyond mechanical effects on lung function, obesity is associated with chronic low-grade systemic inflammation and metabolic dysregulation. Obesity is also consistently linked to gut microbiota dysbiosis, characterized by reduced microbial diversity and altered microbial composition, which may amplify systemic inflammation and influence airway inflammatory profiles (Turnbaugh et al., 2009; Cani et al., 2008; Cani, 2013).

### Allergic diseases

Allergic rhinitis is one of the most common comorbidities of asthma, coexisting in more than 80% of patients and often preceding asthma onset (Leynaert et al., 2000; Bousquet et al., 2008). This close association supports the “one airway” concept, whereby upper and lower airway inflammation share common immunopathological mechanisms. Other atopic conditions, including atopic dermatitis and food allergy, frequently cluster with asthma and reflect an underlying atopic predisposition (Dharmage, Perret & Custovic, 2019; Arrieta et al., 2015).

The gut microbiota plays a crucial role in establishing immune tolerance. Reduced early-life microbial diversity and depletion of beneficial commensal bacteria have been associated with enhanced IgE-mediated immune responses and increased susceptibility to allergic diseases and asthma (Arrieta et al., 2015; Abrahamsson et al., 2014; Sjögren et al., 2009; Cahenzli et al., 2013).

### Chronic rhinosinusitis

Chronic rhinosinusitis, particularly chronic rhinosinusitis with nasal polyps, frequently coexists with moderate-to-severe asthma and is often driven by Type 2 inflammation (Stevens et al., 2017; Michalik et al., 2023). Persistent sinonasal inflammation can exacerbate lower airway disease and contribute to poor asthma control. Emerging evidence suggests that patients with chronic rhinosinusitis exhibit alterations in gut microbiota composition, including reduced abundance of anti-inflammatory and barrier-supporting taxa, indicating a potential role for systemic microbial–immune interactions (Stevens et al., 2017; Michalik et al., 2023).

### Gastroesophageal reflux disease

Gastroesophageal reflux disease is highly prevalent in asthma and exhibits a bidirectional relationship (Tsai et al., 2010). Microaspiration of gastric contents and reflux-induced vagal reflexes can worsen airway inflammation, while asthma-related changes in intrathoracic pressure may promote reflux (Amarasiri et al., 2013). Interestingly, colonization with *Helicobacter pylori*, particularly in early life, has been inversely associated with asthma and allergic diseases, potentially through induction of regulatory immune responses (Wang et al., 2022; Arnold et al., 2011; Chen & Blaser, 2008). Declining prevalence of *H. pylori* infection may therefore contribute to increasing asthma incidence.

## Mechanisms Linking Gut Microbiota and Asthma

### The gut–lung and lung–gut axis

The gut–lung axis refers to bidirectional immune and metabolic communication between the gastrointestinal tract and the respiratory system (Zhang et al., 2025; Budden et al., 2017). Immune cells educated in the gut-associated lymphoid tissue can migrate to the lungs, influencing airway immune responses. Conversely, pulmonary inflammation may alter gut microbial composition through systemic inflammatory mediators (Jiao et al., 2020; Enaud et al., 2020).

Cytokines such as interleukin-6 and interleukin-10 play opposing roles in this axis. Interleukin-6 promotes systemic and airway inflammation, whereas interleukin-10 supports immune tolerance and resolution of inflammation. Imbalance between these pathways may contribute to asthma severity and persistence (Alashkar Alhamwe et al., 2023).

**Figure 1:**
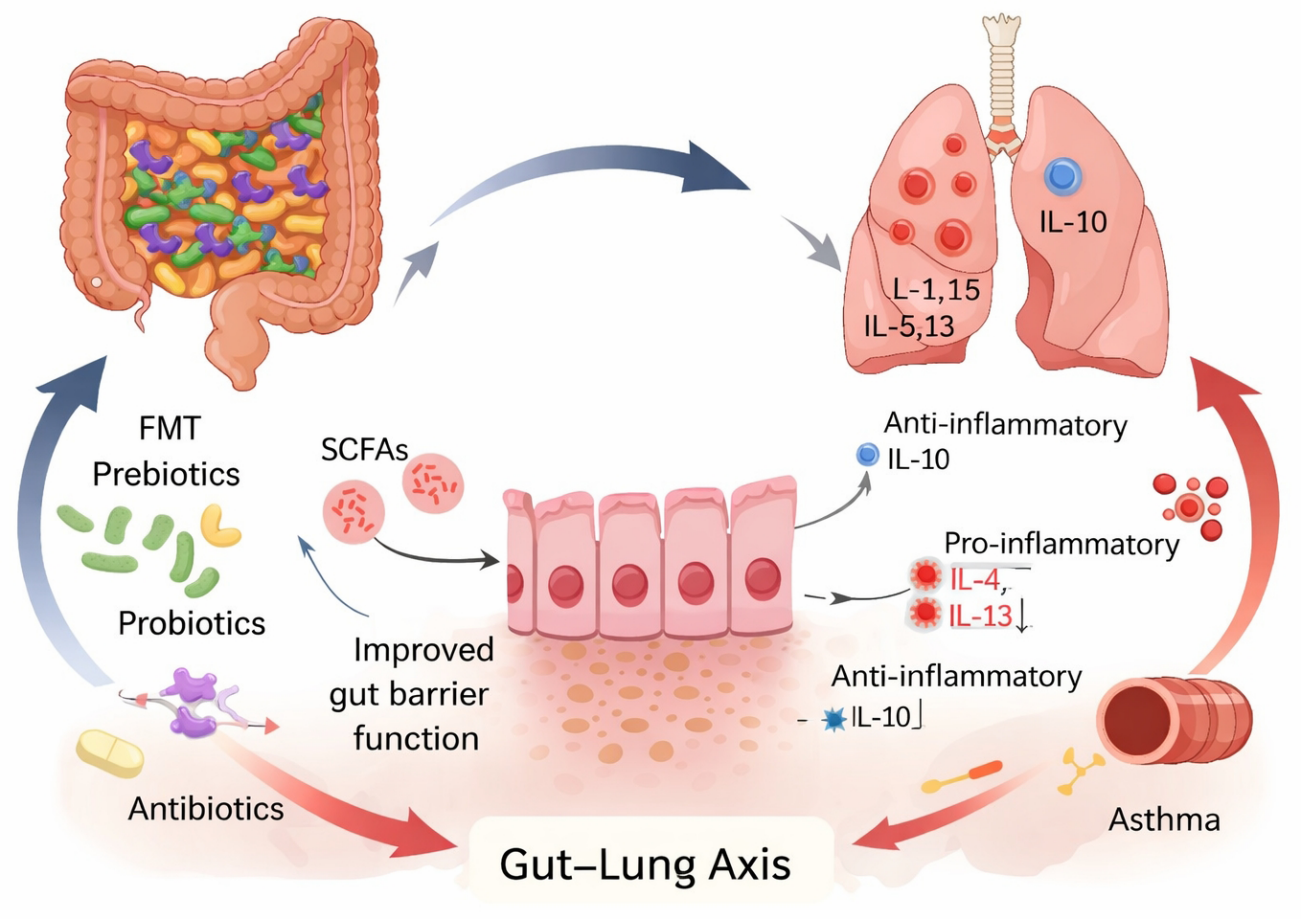
Schematic illustration of the gut–lung axis in asthma. This figure was created by the authors and is an original illustration.

These interactions are summarized in Fig. 1, which illustrates the bidirectional gut–lung axis linking gut microbiota dysbiosis, immune regulation, and asthma with its common comorbidities.

Dietary components (*e.g.*, dietary fiber, oligosaccharides, and polyphenols) are fermented by the gut microbiota to produce short-chain fatty acids (SCFAs), which modulate systemic and pulmonary immunity through regulatory T cell (Treg) induction, histone deacetylase inhibition, and cytokine regulation (*e.g.*, IL-10 and IL-6). Gut microbiota dysbiosis leads to impaired epithelial barrier integrity, increased microbial translocation, and chronic low-grade inflammation. Immune cells and microbial metabolites traffic bidirectionally between the gut and lungs, influencing airway inflammation, asthma severity, and the development of comorbidities including obesity, allergic diseases, chronic rhinosinusitis, and gastroesophageal reflux disease.

### Short-chain fatty acids and nutritional interactions

The gut microbiota ferments dietary fiber, oligosaccharides, and polyphenols to produce short-chain fatty acids (SCFAs), including acetate, propionate, and butyrate (Silva, Bernardi & Frozza, 2020; Huffnagle, 2014; Trompette et al., 2014). SCFAs exert immunomodulatory effects by activating G-protein-coupled receptors, inhibiting histone deacetylases, and promoting regulatory T cell differentiation (Liu et al., 2025).

Dietary oligosaccharides and polyphenols act synergistically as microbial substrates, enhancing SCFA production and amplifying anti-inflammatory immune effects. Through these mechanisms, diet–microbiota interactions can influence systemic immunity and airway inflammation (Den Elzen et al., 2025).

### Early-life immune programming

Neonates exhibit a physiological T helper 2–biased immune profile that, under appropriate microbial and nutritional conditions, matures toward a balanced T helper 1 and regulatory T cell phenotype (Strachan, 1989; Gensollen et al., 2016). Early-life microbial exposure is therefore critical for immune maturation and long-term immune tolerance (Harb et al., 2017; Perveen et al., 2021a; Perveen et al., 2021b).

At the molecular level, protein kinase C*ζ* has been identified as an important regulator of immune maturation. Insufficient microbial exposure or early-life dysbiosis may impair this developmental process, increasing susceptibility to asthma and allergic diseases. From a developmental and evolutionary perspective, reduced microbial diversity in modern environments may contribute to long-term immune dysregulation (McDade, 2012).

### Epigenetic regulation

Microbial metabolites and early-life nutrition influence immune development through epigenetic mechanisms, including DNA methylation, microRNA regulation, and chromatin remodeling (Acevedo et al., 2021; van Esch et al., 2020). These processes enable long-term “immune programming,” linking early microbial exposure to persistent alterations in immune function and disease susceptibility. Epigenetic regulation provides a mechanistic bridge between environmental exposures, gut microbiota, and chronic inflammatory diseases such as asthma.

## Influence of the Gut Microbiota on Asthma Comorbidities

Gut microbiota dysbiosis may represent a common pathological link underlying asthma and its major comorbidities. By modulating systemic inflammation, immune tolerance, and metabolic pathways, alterations in the gut microbial ecosystem may contribute to the clustering of obesity, allergic diseases, chronic rhinosinusitis, and GERD in patients with asthma.

The major gut microbiota alterations, immune characteristics, and clinical implications of common asthma comorbidities are summarized in Table 1.

**Table 1:** Gut microbiota dysbiosis in asthma and its common comorbidities.

**Asthma comorbidity**	**Key gut microbiota alterations**	**Dominant immune features**	**Clinical implications**
Obesity	Reduced microbial diversity; altered Firmicutes/Bacteroidetes ratio	Low-grade systemic inflammation; Th17 skewing; reduced Treg activity	Poor asthma control; corticosteroid resistance
Allergic rhinitis/Atopy	Reduced early-life diversity; depletion of *Bifidobacterium* and *Faecalibacterium*	Th2-dominant immunity; elevated IgE	Early-onset asthma; allergic multimorbidity
Chronic rhinosinusitis	Reduced anti-inflammatory taxa (e.g., *Faecalibacterium prausnitzii*)	Persistent type 2 inflammation; impaired mucosal immunity	Severe asthma; frequent exacerbations
GERD	Altered gastric and intestinal microbiota; reduced *Helicobacter pylori*	Neurogenic reflex activation; systemic inflammation	Bidirectional asthma–GERD worsening

## Advances in Microbiota-Targeted Interventions

Growing recognition of the gut microbiota’s role in asthma has prompted interest in microbiota-targeted interventions. Probiotics and prebiotics have been investigated as adjunct therapies, with mixed clinical results likely due to heterogeneity in strains, dosage, and timing of administration.

Dietary modification represents a promising and accessible strategy. High-fiber, plant-rich diets enhance microbial diversity and SCFA production and have been associated with improved asthma outcomes. In contrast, Western-style diets characterized by high fat and low fiber intake may exacerbate inflammation and asthma severity.

Fecal microbiota transplantation has demonstrated efficacy in restoring microbial balance in gastrointestinal diseases and has shown encouraging results in animal models of asthma. However, human data remain limited, and safety considerations currently restrict its clinical application.

An overview of microbiota-targeted intervention strategies, their proposed mechanisms, evidence level, and current limitations is provided in Table 2.

**Table 2:** Microbiota-targeted intervention strategies for asthma and its comorbidities.

**Intervention**	**Proposed mechanisms**	**Evidence level**	**Current limitations**
Probiotics	Restore microbial balance; enhance Treg and IL-10 responses	Mixed clinical evidence	Strain- and timing-dependent effects
Prebiotics/dietary fiber	Increase SCFA production; inhibit inflammatory pathways	Moderate (animal + observational human data)	Dietary adherence variability
Dietary modification	Improve microbial diversity; reduce systemic inflammation	Moderate	Lifestyle confounding factors
Fecal microbiota transplantation	Global microbiota restoration; increase SCFAs	Preclinical/limited human data	Safety and donor selection concerns
Postbiotics	Direct immune modulation without live microbes	Experimental	Lack of large clinical trials

## Conclusion

Asthma is a complex systemic inflammatory disease shaped by interactions between host immunity, metabolism, and environmental exposures. Increasing evidence indicates that gut microbiota dysbiosis plays a central role not only in asthma pathogenesis but also in the development of its common comorbidities.

Through the gut–lung axis, microbial metabolites, immune signaling pathways, and epigenetic mechanisms jointly influence immune homeostasis and airway inflammation. These interconnected processes help explain why asthma frequently clusters with conditions such as obesity, allergic diseases, chronic rhinosinusitis, and gastroesophageal reflux disease.

While mechanisms such as short-chain fatty acid–mediated immune regulation are supported by substantial experimental and clinical evidence, microbiota-targeted therapeutic strategies remain an evolving field. Many interventions show promise in preclinical studies, but robust human data are still limited.

Future research should prioritize well-designed clinical trials, mechanistic precision, and personalized approaches that account for inter-individual microbial variability. A more integrated understanding of diet–microbiota–immune interactions may ultimately enable improved prevention strategies and more holistic management of asthma and its comorbidities.

## Additional Information and Declarations

### Data Availability

The following information was supplied regarding data availability: This is a literature review.
